# Systematic Review of ED-based Intimate Partner Violence Intervention Research

**DOI:** 10.5811/westjem.2015.10.27586

**Published:** 2015-12-08

**Authors:** Esther K. Choo, Amy S. Gottlieb, Marie DeLuca, Chantal Tape, Lauren Colwell, Caron Zlotnick

**Affiliations:** * Warren Alpert Medical School of Brown University, Department of Emergency Medicine, Division of Sex and Gender in Emergency Care, Providence, Rhode Island; † Baystate Health, Springfield, Massachusetts; ‡ Warren Alpert Medical School of Brown University, Providence, Rhode Island; § Brown University, Providence, Rhode Island; ¶ Warren Alpert Medical School of Brown University, Department of Psychiatry and Human Behavior, Butler Hospital, Providence, Rhode Island

## Abstract

**Introduction:**

Assessment reactivity may be a factor in the modest results of brief interventions for substance use in the emergency department (ED). The presence of assessment reactivity in studies of interventions for intimate partner violence (IPV) has not been studied. Our objectives were to identify ED IPV intervention studies and evaluate the presence of a consistently positive effect on the control groups.

**Methods:**

We performed a systematic search of electronic databases for English=language intervention studies addressing IPV in the ED published since 1990. Study selection and assessment of methodologic quality were performed by two independent reviewers. Data extraction was performed by one reviewer and then independently checked for completeness and accuracy by a second reviewer.

**Results:**

Of 3,620 unique manuscripts identified by database search, 667 underwent abstract review and 12 underwent full-text review. Only three met full eligibility criteria; data on the control arm were available for two studies. In these two studies, IPV-related outcomes improved for both the experimental and control condition.

**Conclusion:**

The paucity of controlled trials of IPV precluded a robust evaluation for assessment reactivity. This study highlighted a critical gap in ED research on IPV.

## INTRODUCTION

The emergency department (ED) has been identified as an ideal location to screen for conditions with public health significance, including violence, substance use, mental health disorders, and human immunodeficiency virus risk.^1^ The ED offers access to a large proportion of the U.S. population,^2^ a high prevalence of risky health behaviors,^3^,^4^ and distinctly, a window of higher susceptibility to health messages.^5^

Intimate partner violence (IPV) has been a target of ED interventions for many years, not only because it is the site for injury presentations related to IPV, but also because its prevalence in the ED population is higher than in the general population and in other healthcare settings. However, IPV studies in general, and ED IPV studies in particular, have had a difficult time demonstrating improvement in outcomes after interventions.^6^–^8^ IPV research has been dogged by many known challenges, some inherent to the problem itself, and these have served as explanations for the lack of successful interventions.^9^ First, abuse may manifest in many different ways and affect victims differently, making the study population and relevant outcomes heterogeneous. Change may not occur immediately or in a linear fashion^10^ requiring longer time periods for follow up. Maintaining contact with the target population may be difficult given their circumstances, which may involve social isolation and restricted access to others, including healthcare providers. Finally, interventions themselves (e.g., shelters and separation from the home shared with the abuser) may restrict contact for follow-up measures.

Another possibility for lack of positive IPV intervention studies, however, is “assessment reactivity,”^11^,^12^ the tendency of control subjects to change behaviors solely in response to survey instruments, likely due to increased self-awareness of the behavior and its negative consequences.^8^ Such changes would minimize observed differences between control and test subjects, potentially obscuring the efficacy of an intervention. This phenomenon has been discussed frequently in ED substance use research. Although EDs have employed screening, brief intervention, and referral to treatment (SBIRT) programs to reduce substance use for over 20 years,^13^,^14^ there have been conflicting data on program effectiveness. One theory for the lack of consistent effect shown by SBIRT is that the true effect size has been blunted by assessment reactivity.^15^ Indeed, a number of ED substance use studies have demonstrated marked improvements in control groups.^16^–^18^

Could assessment reactivity influence outcomes of interventions for IPV? While interventions for IPV do not reach the source of the problem (the perpetrator of abuse), they do aim to change the behavior of the survivor in order to improve health outcomes. Many commonly used instruments for measuring IPV include extensive questions about the consequences of abuse, such as negative effects on health and children. Disclosure of prior trauma in general – though not specifically IPV – has been demonstrated to have mental and physical health effects, even including enhancement of immune function, potentially due to the cathartic nature of the disclosure.^19^–^23^ Investigators have described striking responses to assessments among IPV survivors, including strong emotional reactions to divulging IPV, epiphanies about the nature of their relationships, and determination to seek help from domestic violence agencies and to use safety behaviors in the future.^24^,^25^ Further, there is some evidence that women are more susceptible to assessment reactivity,^26^ making its presence even more likely in IPV interventions, which are typically targeted to women.

Understanding the effect of assessment on IPV studies has potential implications for both clinical care and research.^27^ In clinical practice, skepticism has dogged IPV screening recommendations; the United States Preventive Services Task Force did not advise routine screening for IPV until 2013,^28^ citing a lack of evidence for their health benefits and safety. A known assessment effect might reframe incremental or borderline intervention effects in existing studies, bolstering the argument for screening. For researchers, a known or suspected assessment effect may prompt study design accommodations. For example, the Solomon four-group design, in which participants within control and intervention groups are randomized further into assessed and unassessed arms, acknowledges the potential for assessment to influence outcomes^29^ and has been used to evaluate the presence of this effect in a variety of intervention studies involving health behaviors.^30^–^33^

To date, the phenomenon of assessment reactivity has not been examined in interventions for IPV in the ED. The objectives of this study were to perform a systematic literature review to identify ED-based studies comparing an intervention and control arm and to evaluate studies for consistent evidence of improvement in the control arm, which would substantiate the presence of assessment reactivity.

## METHODS

### Search Strategy

A medical research librarian worked with the research team to develop a systematic search strategy, including English-language studies published during or after 1990. The search was conducted in 13 databases. The team also reviewed ClinicalTrials.gov and references of all included articles to identify other potentially relevant studies. Search terms included the following: Emergencies, Emergency Service, Emergency Medicine, Accident and Emergency, Casualty; Trauma Ward; Emergency Department; Domestic Violence, Intimate Partner Violence, Partner Abuse, Spouse Abuse, Battering, Battered Women. The searches were completed between June and October 2013. Data extraction and synthesis were conducted from October to December 2013.

### Study Selection

We included studies that provided any IPV initiative, whether screening with physician notification or a specific, well-defined intervention, that compared a “control” with a “test” group, and had pre- and post-assessment to determine clinically significant IPV-related outcomes, as defined by investigators. We excluded studies with only acceptability or attitudes as outcome measures.

### Data Abstraction and Analysis

Investigators reviewed titles to identify potentially eligible articles and to eliminate duplicates across databases; secondary review was performed on a subset of titles from the full list of titles to verify the quality of the initial screen. During an initial training period, titles were reviewed as a group and any lack of consensus was resolved through discussion. Thereafter, group review occurred periodically throughout the study to maintain fidelity to research criteria.

Two investigators reviewed the abstracts of each article retained after title review. If at least one investigator felt a study was potentially eligible based on abstract review, the full manuscript was retained for review. Two investigators independently read the manuscripts to determine if each study met eligibility criteria. Any discrepancies in opinion were resolved by discussion with the senior investigator.

Using a standardized abstraction form, one investigator extracted data on study design, study population, definition of IPV, nature of the intervention, assessments, and outcomes. Accuracy of information was confirmed by a second investigator. Two investigators independently computed quality scores for each study using Jadad criteria.^34^ The Jadad scale was selected for use given its incorporation of common sources of bias in randomized controlled trials, its established validity and reliability,^35^ and its ease of use. Given the small number of studies that met full eligibility criteria, we did not attempt to perform a pooled meta analysis, but examined studies descriptively only.

## RESULTS

Of 3,620 unique studies initially identified by the search, title review yielded 667 abstracts for review: 12 met criteria for full text review. Of these 12, six were purely observational, without a studied intervention, one did not have a comparison group and two did not include an ED site, leaving three studies that met full criteria for inclusion in the current study. These studies’ characteristics were summarized in the Table.

In Study 1,^36^ adult women with IPV were recruited to a randomized trial of an Emergency Department Victim Advocacy (EDVA) protocol. The intervention arm received a session with a victim advocate, which involved empathic support, empowerment counseling, education about the dynamics of abuse, safety assessment, safety planning, linkage with community resources, and support and assistance with follow up. The comparison group received empathic support, safety assessment, and linkage with community resources from a social worker. Outcomes included readiness to end the abusive relationship, use of community resources, safety planning, occurrence of abuse and mental health. All outcome measures in both control and intervention arms demonstrated improvements as a function of time, not treatment condition.

Study 2 recruited adult women patients from the ED, family practice, or obstetrics/gynecology clinic.^27^ Shifts or days were randomized to systematic screening for IPV. During screening times, research assistants placed positive questionnaires in the clinical charts. Any discussion or referrals were at the discretion of the treating provider. On control days, participants completed screening after their clinical encounter. Primary outcomes included occurrence of IPV and overall quality of life. Authors reported that the “trajectory of IPV recurrence risk was downward” and quality-of-life scores improved for all participants; the improvement appeared more rapid in the intervention group, but this effect disappeared in the more robust dataset with multiple imputation of missing data.

Study 3^37^ recruited African-American women from an adult ED to participate in a randomized trial of an educational intervention for high-risk health problems. Eligible participants took a computerized health screening survey. Those who screened positive for IPV, alcohol or drug abuse or cigarette smoking were randomized to intervention or control groups. The intervention group received brochures tailored to their health issues, reviewed with them in person by a research assistant. The control group received brochures for neighborhood health clinics. Primary outcomes were contact with social support agencies and harm-reduction actions, such as making a smoking cessation plan. Although the study reported that the intervention group was more likely to contact service agencies, results stratified by study assignment were not available.

None of the studies used minimal or no-assessment groups at baseline.

## DISCUSSION

In 1985, when Surgeon General C. Everett Koop cast light on the public health significance of domestic violence, the problem moved solidly into the domain of healthcare professionals. EDs provided some of the earliest clinically-based research data on the prevalence, health outcomes and co-occurring disorders of violence against women.^38^–^40^ EDs have also been the site for testing large-scale computer-based screening in the waiting room, demonstrating that disclosure may be easier through a computer-based medium.^41^ However, our field has published few clinical trials employing rigorous methods to examine the effect of screening and/or interventions for IPV on health outcomes of women.

Consistent with prior research, the present study used a liberal definition of “intervention” by including screening-only initiatives. We also accepted studies conducted in a variety of settings, as long as EDs were included. Even so, we identified only three controlled clinical trials of IPV initiatives in the ED. Our evaluation of assessment reactivity was limited primarily by this dearth of IPV studies. Additionally, one of the studies included provided no individual outcomes for the control group, so assessment effect could not be examined. Of the two remaining studies, both described outcome improvements in the control condition. One, however, provided an enhanced control condition, so the positive effect on controls was unlikely to be purely from assessments received.

It is important to note the high loss-to-follow-up rates in the included studies, ranging from 49 to 78%. Loss to follow up is a common problem in high-risk populations^42^,^43^ and may bias results, potentially obscuring a positive outcome, e.g., if those in the intervention group are more likely to stay engaged and report ongoing abuse. Therefore, loss to follow up not only limits the conclusions that can be drawn from the described studies, but also serves as an alternate explanation to assessment reactivity for the lack of intervention effect observed in IPV studies.

Our investigation of assessment reactivity in the study of IPV in the ED was neither able to confirm nor refute its presence. As clinical trials of IPV emerge, it will be important to consider the potential impact of such study design factors on the measurement of outcomes. IPV interventions share many similarities to substance-use interventions, including needing to overcome significant reserve and shame around disclosure and the opportunity to capitalize upon the ED “teachable moment”^3^,^44^ to stimulate action around high-risk health issues. However, these factors also increase the possibility that simply divulging the problem can lead to change – a wonderful thing for those prioritizing brief, scaled-down interventions in this setting but critical to the understanding and interpretation of research results.

Our study is limited by its narrow inclusion of ED studies only. While this decision was made to target the setting where assessment reactivity seemed most likely to be influential, and in order to inform ED-based investigators developing future IPV interventions, it did limit the number of studies available for analysis. Future systematic reviews may include IPV interventions in other healthcare settings, including primary care and obstetrics and gynecology clinics.

Although we were not, ultimately, able to study assessment reactivity, our efforts did highlight a critical gap in emergency medicine literature. IPV intervention studies are needed, and they should meet the standards of other clinical trials, going beyond observational or quasi-experimental designs. Our study underscores that there is very little known about the effect of simply asking questions about IPV in the ED, let alone about the effect of well-developed, standardized brief interventions and referrals to treatment. It is important for those arguing for or against IPV screening to remember that the absence of evidence is not evidence of absence.

## Figures and Tables

**Table:** Randomized controlled trials of emergency department (ED) intimate partner violence (IPV) Interventions.

Study (First author, year)	Target population	Number of enrolled participants (% retained)	Intervention	Control	Primary outcomes for control condition
Study 1 (Hyman, 2001)	Women >18 years old screening positive upon nursing query for IPV in the ED (≥2 positive responses on a Domestic Safety Assessment)	102 (51% completed 3–4 month follow up in person)	Emergency Department Victim Advocacy (EDVA)	Standard Social Service (SSSI)	Readiness: Mean scores for inactive stages of change decreased and those for active stages of change increased for all participants as a function of time, not treatment condition. Community Resources: Mean number of resources used declined in the control group (which had higher resource utilization at baseline) but remained higher than for the intervention group. Safety Behaviors: All participants increased safety behaviors over time, regardless of treatment condition. Abuse: Index of Spouse Abuse (ISA) total score decreased as a function of time, not treatment condition. Mental Health: Global Severity Index (GSI) decreased as a function of time, not treatment condition; Impact of Event Scale (IES) and Structured Clinical Interview for DSM-IV (SCID) scores decreased for all participants.
Study 2 (MacMillan, H, 2009)	Women 18–64 presenting to an ED, family practice, or OB/GYN clinic completing a written self-interview screen	707 (57% screened, 59% nonscreened completed 18 month follow up by in-person assessment and written self-interview)	Systematic IPV screening (using the Women’s Abuse Screening Test, WAST); clinician informed of positive screens.	Screening at the discretion of the treating clinician.	IPV Recurrence: Declined over time: 64% at 6 mos; 59% at 12 mos; 53% at 18 mos Quality of Life Scores: Improved over time: 50.6 +/− 17.2 at baseline; 50.5 +/− 17.9 at 6 mos; 52.5 +/− 18.0 at 12 mos; 52.7 +/− 17.9 at 18 mos
Study 3 (Houry, D, 2011)	African-American women 21–55 presenting to an ED who completed a computerized health screening survey	322 (22% completed 3 month follow up with computer screening and in-person assessment)	Brochures tailored to relevant health issues provided to patients and reviewed with them by research assistants.	Brochure with information about clinics in the area.	Not available.
